# Pelvic arteriovenous malformation (AVM) with recurrent hematuria: A case report

**DOI:** 10.1016/j.ijscr.2023.108701

**Published:** 2023-08-22

**Authors:** Iraj Nazari, Mohammad Amin Zargar, Pegah Panahi, Seyed Mohammad Amin Alavi

**Affiliations:** a Department of General Surgery, School of Medicine, Ahvaz Jundishapur University of Medical Sciences, Ahvaz, Iran; b Faculty of Medicine, Ahvaz Jundishapur University of Medical Sciences, Ahvaz, Iran.

**Keywords:** Arteriovenous malformation, Aneurysm, Angiography, Embolotherapy, Pelvis, Surgery

## Abstract

**Introduction:**

Arteriovenous malformation (AVM) leads to a direct connection between arterial and venous networks, in which capillary branches are not involved. Pelvic AVM is a benign and rare condition causing severe pain, hematuria, and rectal or vaginal bleeding.

**Case presentation:**

A 36-year-old woman presented with five months history of hematuria. Her medical history was unremarkable, and laboratory findings were all within normal ranges. Abdominopelvic computed tomography (CT) scan revealed a vascular mass in the left lateral pelvis that extended to the bladder neck and was suggestive of an AVM. The patient underwent a laparotomy for the resection of AVM. The first angiography revealed an AVM in the left internal iliac artery. The patient underwent embolization with coil and gel foam. The second angiography revealed complete obstruction of the left internal iliac artery due to multiple coils and AVM of the right internal iliac artery (RIIA), embolized with glue and lipiodol. A week later, venography revealed another left iliac vein malformation embolized with foam sclerotherapy. Forty days later, the third angiography revealed another AVM in the right iliac artery, embolized with three vials of polyvinyl alcohol (PVA). Following two months of follow-up, the symptoms did not return.

**Discussion:**

The present study reported a rare case of recurrent pelvic AVM causing painless hematuria in a female patient. The lesion was treated with several angioembolization sessions.

**Conclusion:**

Angioembolization is one of the main therapeutic options for AVM. Appropriate material should be precisely chosen for AVM embolization regarding the AVM's location, size, and condition.

## Highlights

• The present study describes the treatment of a rare type of recurrent pelvic arteriovenous malformation (AVM) using several angiography-based embolization sessions.
• Angiography can be an effective diagnostic modality for pelvic AVM.
• Appropriate material should be precisely chosen for AVM embolization regarding the location, size, and condition of the AVM.

## 1 Introduction

As an abnormality of the development of capillaries, arteriovenous malformation (AVM) leads to a direct connection between arterial and venous networks, in which capillary branches are not involved [1]. Typically, these lesions are low-resistant and high-flow vascular channels with different sizes and locations [2,3].

The occurrence of AVMs in the pelvis is quite rare. Moreover, pelvic AVMs are usually benign lesions. However, they may lead to severe pain, hematuria, and abnormal vaginal or rectal hemorrhage depending on the related organs. Also, the AVMs can affect the adjacent structures [1,4]. It has been shown that selective angiography is the best diagnostic option for pelvic AVMs. Moreover, computed tomography (CT) scan has been proven to be beneficial as well [5]. The present study aims to report a case of pelvis AVM who is a 36-year-old woman presenting with painless hematuria. This case report has been reported in line with the SCARE 2020 Criteria [6].

## 2 Case presentation

The present case was a 36-year-old woman presenting with gross hematuria for the last 5 months. The hematuria was intermittent and included large clots. The patient did not report any remarkable medical history, underlying diseases, current illness, ongoing medication use, relevant family history, and any surgical history involving the pelvis and urinary system. Moreover, she did not report any cigarette smoking or alcohol consumption. Regarding obstetric history, she reported 4 previous caesarian sections, with the last one being 2 years ago.

No abnormal finding was reported in the physical examination. Moreover, laboratory investigations showed normal Complete Blood Count (CBC), bleeding time, and coagulation tests, except for a low hemoglobin level (7.2 g/dl).

**Fig. 1:**
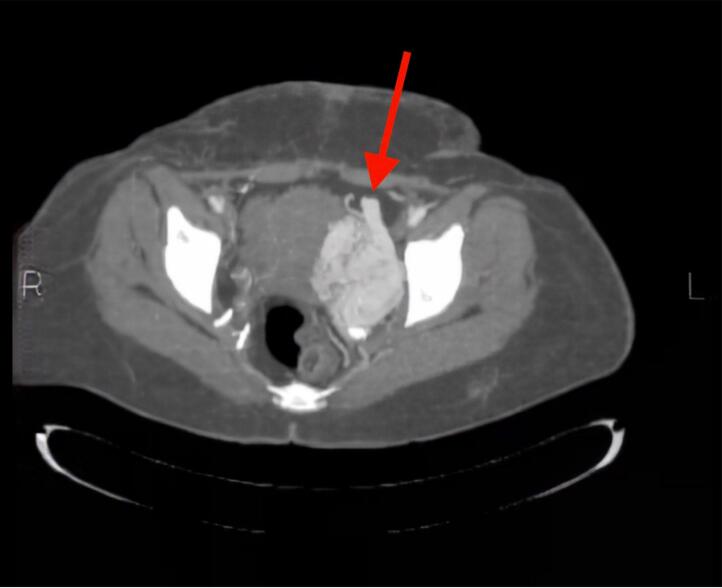
Abdominopelvic CT scan revealing a vascular mass in the left lateral pelvis that extended to the bladder neck highly suggestive of an AVM.

The patient underwent cystoscopy twice by the urology department, and several blood clots were discharged from the bladder. However, no abnormality was found in the bladder and urethra, except for a small urethral ulcer. Moreover, an abdominopelvic CT scan with IV contrast was ordered, revealing a vascular mass in the left lateral pelvis that extended to the bladder neck and was suggestive of an AVM (Fig. 1). Then, the patient underwent a laparotomy for the resection of AVM by the urology team. Due to the AVM's low access, the urologist requested a vascular surgery consult in the surgery theatre.

The decision to perform a post-operative therapeutic angiography was made regarding the retroperitoneal exploration by the vascular surgeon during the laparotomy (Video and Still 1). For the angiography, a 6 French (F) guage catheter was inserted retrogradely through the left femoral artery into the aorta. The aortogram revealed excessive blood flow through the left internal iliac artery, which was the site of the AVM. Then, the vascular surgeon crossed the catheter, entered the left internal iliac artery, and started to embolize the nidus from the distal part of the artery using coil and gel foam. There was no obvious pathology in the (RIIA) at the time of the first angiography. Afterward, the patient was discharged and was symptom-free for weeks. However, she returned with severe hematuria later.

The patient underwent a diagnostic angiography again in another center and was diagnosed with complete obstruction of the left internal iliac artery due to multiple coils. Moreover, an AVM was observed on the right side of the pelvis that was related to the RIIA (Video and Still 2). The procedure was similar to previous angiography but from the contralateral side. The observed AVM was embolized with a combination of glue and lipiodol. A week later, the hematuria of the patient was not stopped. Thus, she underwent venography, revealing another malformation related to the left iliac vein (Video and Still 3). The vascular surgeon inserted the catheter through the right femoral vein and then entered to left internal iliac vein (LIIV). A balloon was inserted into the proximal vein, and the observed AVM was embolized with foam sclerotherapy (fibrovein 3 %) and 97 % alcohol.

Following 40 days after the last angiography, the patient returned with gross hematuria again. Therefore, another angiography (Video and Still 4) was performed, in which the vascular surgeon initiated the angiography from the left femoral artery, and then the catheter was inserted into the right internal iliac artery through the left internal iliac artery. Finally, the surgeon inserted the catheter into the ovarian artery and embolized the nidus with three vials of polyvinyl alcohol (PVA). Following four months of follow-up, the symptoms did not return.

## 3 Discussion

As a vascular abnormality, AVM is a snarled tangle of arteries and veins connected without capillaries, thereby disturbing organ perfusion. It has been shown that the global incidence and prevalence of AVMs are 1 and 10 cases per 100,000 individuals, respectively [[7], [8], [9]]. Moreover, these vascular lesions can be congenital or acquired. Congenital AVMs originate in the embryologic blood vessels due to an unpremeditated defeat in the focal vascular development occurring in the 4–10 weeks of gestation. These lesions are found in multiple feeding vessels. However, acquired AVMs are related to one or a few feeding vessels and are caused by trauma, tumors, or surgery [8,10].

In most cases of acquired AVM, an iatrogenic history is present, such as a previous cesarean section or Dilation and Curettage (D & C). Many patients with these lesions are women of reproductive age with a history of spontaneous abortion, gestational trophoblastic disease, or infection without any family history. AVMs develop during the process of tissue repair, wherein an improper connection forms between an artery and veins. However, the majority of AVM cases are sporadic and are due to genetic mutations, such as the mutations in ENG and ALK-1 in the hereditary hemorrhagic telangiectasia, RASA-1 in capillary malformation/arteriovenous malformation (CM-AVM) syndrome, or PTEN in Bannayan-Riley-Ruvalcaba or Cowden syndromes [[10], [11], [12]].

The characteristic presentations of pelvic AVMs are abdominal or pelvic discomfort and pain, rectal pain, tenesmus, low back or sciatic pain (rarely), and genitourinary manifestations, such as hematuria, hydronephrosis, hemospermia, impotence, and orchitis. Moreover, it has been shown that large AVMs may lead to shunts causing congestive heart failure [13]. However, 20 % of the patients are asymptomatic [11].

Numerous noninvasive diagnostic methods can be used for diagnosing pelvic AVMs, including contrast-enhanced CT scan, Magnetic Resonance Imaging (MRI), and color Doppler ultrasound [14]. Nonetheless, angiography, which is an invasive diagnostic modality, is the gold standard for differentiating between primary blood circulation and vascular malformation, detecting a nidus, evaluating the magnitude of arteriovenous shunting, and venous drainage [15,16].

Pelvic AVMs are generally difficult to treat because of their complex vascular nature [17]. Surgical procedures, such as catheter-guided procedures or stereotactic radiosurgery, are the main options for AVM treatment and can be used for the resection, embolization, or radiation of the AVMs to reduce the associated risks. However, the occurrence of reperfusion due to recanalization, prior unidentified feeding, or adjacent arteries leads to AVM recurrence in about 25 % of the patients one year after the intervention. If the surgical interventions cannot be presumed to be safe or appropriate, medical treatments are preferred for multiple or diffuse minor AVMs [3].

There are multiple treatment options for pelvic AVM, including ligation of the afferent artery, excision, and embolization. Nevertheless, it has been shown that the surgical method is typically ineffective and fraught with complications such as hemorrhage injury to adjacent organs, and a possibility of recurrence [18]. The first transcatheter embolization was carried out in the early 1970s [19]. During early 1980s, embolization was established as the treatment of choice for pelvic AVMs [20]. Due to the lower morbidity, mortality, and invasiveness, embolization is the treatment of choice for pelvic AVM [18]. A study by Cansaran et al. in 2021 reported a case of AVM around the bladder that was first mistaken for an appendicular abscess and was excised by open surgery. Thus, the authors suggested open surgery as another therapeutic method for AVMs [21]. However, insufficient removal of the arteriovenous malformation (AVM) lesion or the occlusion of the arterial input might lead to the recurrence or rapid progression of the AVM [22]. In most cases, embolization is quite effective. However, the efficacy of surgery is debated. Some researchers have suggested a surgical excision following embolization in patients with AVMs without visceral invasion [20]. Moreover, a study by Kass in 2022 investigated a case of AVM resection during a 4-h robot-assisted surgery, suggesting this method as a reasonable treatment for pelvic AVM in case of unsuccessful embolization [23].

A systematic approach might be applied to ascertain the most suitable effective agent. Three critical factors can help the physician to choose the proper agent: a) the size of the vessel, b) the need for temporary or permanent occlusion, and c) the viability of the organ after the embolization. It should be mentioned that every vessel that is visible on angiography is classified as large. In general, there is a negative correlation between particle size and the probability of ischemic events. In this manner, coils plus gel foam can be utilized for permanent occlusion of large vessels. For the small vessels, including AVMs, where tissue death is desirable, proper agents are sclerosant liquid (absolute alcohol) and glues [19,24]. As the vessel was visible in the first angiography, the vascular surgeon chose a more prominent agent (coil and gel foam). However, after several attempts for the occlusion and recurrence of the AVM, the vascular surgeon decided to utilize smaller agents (alcohol and glue) to embolize the AVM.

## 4 Conclusion

The present study reported a case of pelvic AVM causing painless hematuria in a female patient. The lesion was treated with several angioembolization sessions. Angioembolization is one of the main therapeutic options for AVM. However, it is of utmost importance to inform the patient about the nature of the disease and the possibility of several operations because the patient's cooperation is a necessity for achieving a definitive cure. Another important factor in treatment success is the use of appropriate material depending on the location, size, and condition of the AVM. Embolization materials can be permanent, such as coils, or temporary, such as gel foam. Temporary materials are mostly considered in cases with a surgical plan following embolization due to absorption of the material and AVM recurrence.

The following are the supplementary data related to this article.

Video and Still 1 The first angiography showed AVM in the left internal iliac artery. Video and Still 1

Video and Still 2 Second angiography revealed an AVM on the right side of the pelvis related to the right internal iliac artery. Video and Still 2

Video and Still 3 Venography showed malformation related to the left iliac vein. Video and Still 3

Video and Still 4 The third angiography revealed another AVM in the right iliac artery the right iliac artery. Video and Still 4

## Funding statement

No sources of funding were declared for this study.

## Ethics approval statement

Permission from the ethics committee was not required for case reports at the institution where the research was carried out.

## Patient consent statement

Written informed consent for publication of the case report has been signed by the patient and is available upon request from the editors.

## CRediT authorship contribution statement

Iraj Nazari: Involved in the conception and design of the study, revising of the article, and final approval of the version to be submitted and also involved in direct management of the patient.

Mohmmad Amin Zargar: Involved in the conception and design of the study, drafting and revising of the article, and final approval of the version to be submitted. Also involved in direct management of the patient.

Pegah Panahi: Involved in the design of the study, drafting and revising of the article, and final approval of the version to be submitted.

Seyed Mohammad Amin Alavi: Involved in the design of the study, drafting and revising of the article, and final approval of the version to be submitted.

Guarantor: Mohammad Amin Zargar.

## Declaration of competing interest

The authors have no conflict of interest to declare.

## Data Availability

The data supporting the findings of this study are available upon request from the corresponding author.

## References

[1] Kaplan T., Altuntas B., Ceran S., Sadi Sunam G. (2009). Unusual location of arteriovenous malformation; posterior mediastinum. Interact. Cardiovasc. Thorac. Surg..

[2] Mencia M.M., Cassie P., Beharry A., Budhoo E. (2022). En bloc surgical excision of an arteriovenous malformation in the foot: a case report and review of the literature. J. Orthop. Case Rep. India.

[3] Schimmel K., Ali M.K., Tan S.Y., Teng J., Do H.M., Steinberg G.K. (2021). Arteriovenous malformations—current understanding of the pathogenesis with implications for treatment. Int. J. Mol. Sci..

[4] Aymard A., Bisdorf A., Saint-Maurice J.-P., Labeyrie M.-A., Houdart E. (2019). Malformations arterio-veineuses de l’abdomen et du pelvis: diagnostic et indications thérapeutiques. Presse Med. [Internet].

[5] Suzuki K., Tanaka N., Ebine T., Momma T. (2012). Pelvic congenital arteriovenous malformation diagnosed by transrectal ultrasonography: a case report. Can. Urol. Assoc. J..

[6] Agha RA, Franchi T, Sohrabi C, Mathew G, Kerwan A, Thoma A, et al. The SCARE 2020 guideline: updating consensus Surgical CAse REport (SCARE) guidelines. Int. J. Surg. [Internet]. 2020;84:226–30. Available from: https://www.sciencedirect.com/science/article/pii/S1743919120307718. 10.1016/j.ijsu.2020.10.034 33181358

[7] Park J.-M., Park Y.-K., Chang S.-G. (2005). Arteriovenous malformation of the urinary bladder: treated by transurethral resection. Int. J. Urol. Off. J. Jap. Urol. Assoc..

[8] Fayad L., Hazirolan T., Bluemke D., Mitchell S. (2006). Vascular malformations in the extremities: emphasis on MR imaging features that guide treatment options. Skeletal Radiol. [Internet].

[9] National Organization for Rare Disorder (2013). Arteriovenous Malformation. https://rarediseases.org/rare-diseases/arteriovenous-malformation/#affected.

[10] Annam A. (2018). Female pelvic vascular malformations. Semin. Intervent. Radiol..

[11] Bekci T., Yucel S., Turgut E., Soylu A.I. (2015). Giant congenital pelvic AVM causing cardiac failure, diplegia, and neurogenic bladder. Pol. J. Radiol. Poland.

[12] Smith E.S., Gala R.B. (2015). Successful management of cesarean scar pregnancy complicated by an arteriovenous malformation. Ochsner J..

[13] Game X., Berlizot P., Hassan T., Joffre F., Chokairi S., Houlgatte A. (2002). Congenital pelvic arteriovenous malformation in male patients: a rare cause of urological symptoms and role of embolization. Eur. Urol..

[14] Chen S.-Q., Jiang H.-Y., Li J.-B., Fan L., Liu M.-J., Yao S.-Z. (2013). Treatment of uterine arteriovenous malformation by myometrial lesion resection combined with artery occlusion under laparoscopy: a case report and literature review. Eur. J. Obstet. Gynecol. Reprod. Biol..

[15] Yuzaki I., Aramaki-Hattori N., Tamura M., Torikai H., Okabe K., Sakai S. (2020). Arteriovenous malformation on the sole of the foot treated successfully by embolization. Radiol. Case Rep. Netherlands.

[16] Cura M, Elmerhi F, Suri R, Bugnone A, Dalsaso T. Vascular malformations and arteriovenous fistulas of the kidney. Acta Radiol. [Internet]. 2010 Mar 1;51(2):144–9. Available from: 10.3109/02841850903463646. 20092371

[17] Kishino M., Nishida K., Kimura K., Takahashi M., Nakaminato S., Kume H. (2020). Paravesical space arteriovenous malformation as a specific subgroup of pelvic vascular anomaly: a case series and review of literature. Jpn. J. Radiol..

[18] Huang Y., Liu X., Qian H. (2022). A male congenital pelvic arteriovenous malformation diagnosed by abdominal ultrasound: a case report and literature review. Front. Surg. Switzerland.

[19] Lubarsky M., Ray C.E., Funaki B. (2009). Embolization agents-which one should be used when? Part 1: large-vessel embolization. Semin. Intervent. Radiol..

[20] Diwan R.V., Brennan J.N., Selim M.A., McGrew T.L., Rashad F.A., Rustia M.U. (1983). Sonographic diagnosis of arteriovenous malformation of the uterus and pelvis. J. Clin. Ultrasound.

[21] Cansaran S., Atay N.I., Pelin A.K., Celayir A.C., Moralioğlu S., Koc N. (2021). A unique case: arteriovenous malformation of the urinary bladder wall. West Indian Med. J..

[22] Do Y.S., Kim Y.-W., Park K.B., Kim D.-I., Park H.S., Cho S.K. (2012). Endovascular treatment combined with emboloscleorotherapy for pelvic arteriovenous malformations. J. Vasc. Surg..

[23] Kass S.L., McKeever D.N., Mama S.T. (2021). Minimally invasive approach to pelvic arteriovenous malformation (AVM) refractory to embolization. J. Minim. Invasive Gynecol. [Internet].

[24] Lubarsky M., Ray C., Funaki B. (2010). Embolization agents-which one should be used when? Part 2: small-vessel embolization. Semin Intervent Radiol..

